# X-Ray Treatment of Acne

**Published:** 1903-07

**Authors:** Henry R. Slack

**Affiliations:** President Georgia Pasteur Institute, Atlanta; Superintendent LaGrange Sanatorium, LaGrange, Ga.


### X-RAY TREATMENT OF ACNE.*

BY HENRY R. SLACK, PH.M., M.D.,

President Georgia Pasteur Institute, Atlanta; Superintendent LaGrange Sanatorium, LaGrange, Ga.

Probably there is no non-malignant disease that causes more anxiety and annoyance, to both patient and physician, than a stubborn case of acne.

Just as the ambitious youth approaches manhood and begins to stroke with pride a downy upper lip, he notices a blackhead, then a pimple on his face. He thinks at first it is nothing, but when the number increases he is convinced that he needs a blood purifier. He purchases some patent nostrum recommended in the papers for such trouble, but to his chagrin the medicine does no good, and in most cases makes him break out worse. He then consults a physician, and we all know how wearisome many of these cases become. If acne causes anxiety and heart-burnings in the youth, how much greater the suffering endured by the lovely girl just about to graduate. The assurance that the disease will likely pass away in the course of a few years is to her cold comfort.

*Read before the Medical Association of Georgia, April 17, 1908, Columbus, Ga.

There is no need of my discussing before this body the etiology and pathology of acne, since the diagnosis of a well-marked case presents no difficulty. It is the treatment that so often disappoints us as well as our patients.

Knowing two effects produced by X-rays, that of checking pus formation and the atrophy produced in the follicles, suggested its use in treating this troublesome condition of the skin.

The results I have obtained have been very gratifying and are sufficiently encouraging to justify reporting my cases to this association.

CASE 1.—G. Y., aged eighteen, male, brunette, student. General health good, except slight post-nasal catarrh. Has been troubled with acne for two years. Presented himself for treatment August 30, 1902. His forehead, cheeks and neck were covered with thirty-five large inflammatory papules and pustules. He was exposed to X-rays for eight minutes at nine inches, using a soft tube. He was given treatment alternate days until September 6, five in all, when he left for college. He then had only fourteen small papules. Did not see him again until Thanksgiving Day. He had received no further treatment and face was quite smooth.

CASE 2.—J. M., aged nineteen, male, blonde, manager telephone. General health always good except had trouble with throat previous winter and tonsils had been removed. Now has well-marked case of acne. Counted fifty-four papules on forehead, cheeks, nose and neck. He had trouble for eighteen months. Gave him first treatment September 30, 1902, using soft tube for eight minutes at nine inches. Treated three times a week for four weeks. After first week number reduced to thirty, by the end of second week to sixteen, and by the end of third week to six. When I stopped treatment his face was perfectly smooth. I noticed in this case after each treatment face became very oily. He has remained free from pustules ever since.

CASE 3.—Miss A. L. S., aged eighteen, blonde, college girl. Mother had some trouble. General health good. Has had acne for eighteen months. Present attack worse than any ever had before. Has been taking patent medicines freely and using soaps and salves in abundance, but the pustules are discharging freely and leaving ugly pits. March 27, 1903, gave first exposure to X-rays, using soft tube for seven minutes at six inches. Have given three treatments a week since. At this time, April 13, she has had eight exposures and only has two pustules on right cheek.

CASE 4.—Miss R. M., aged sixteen, brunette, college girl. Family history negative. General health good. Face been bumpy for sixteen months despite all treatment. March 27, 1903, had forty-four papules and pustules on forehead, cheek and nose, besides numerous ones on neck and arms. Gave exposure March 27 and three times a week since, using soft tube eight minutes six inches. After the fourth exposure all the pustules on the neck and arms were gone, though they were treated through the clothing, and the number on her face had reduced to sixteen. At this time, April 13, there are only six small papules on the forehead, while cheeks and chin are free.

The only effects noticed from the X-rays were a slight hyperemia and pigmentation of the skin of about the same intensity as that produced by a week's stay at the seashore, and in case 2 the unctuous feeling of the surface.

I have used the X-rays successfully in treating two epitheliomas of the lower lids, one of lower lip and in three carcinomas of the breast. Two of these were pronounced inoperable. In one the knife might have been used, but as her mother, after two operations, had died from cancer of the breast, she preferred trying X-rays. Have also gotten satisfactory results in two cases of tubercular ulcers. One case of recurrent carcinoma of the breast, after two operations, was treated. The neoplasms reduced in size and pain was relieved, but not cured. One case of carcinoma of the lip apparently healed, but metastasis occurred on the gum and did not yield to treatment.

On the whole my experience with X-rays has been very satisfactory. It is not a "cure-all," but is a valuable therapeutic agent that should always be tried before cases are given up as hopeless.

LITERATURE.

Pusey, *Jour. Cutan. and Gen.-Urin. Dis.*, May, 1902. Campbell, *Jour. Amer. Med. Asso.*, August 9, 1902.

